# Determinants of pre-lacteal feeding practices in urban and rural Nigeria; a population-based cross-sectional study using the 2013 Nigeria demographic and health survey data

**DOI:** 10.4314/ahs.v17i3.11

**Published:** 2017-09

**Authors:** Anselm Shekwagu Berde, Siddika Songul Yalcin, Hilal Ozcebe, Sarp Uner, Ozge Karadag Caman

**Affiliations:** 1 Africa Unit for Transdisciplinary Health Research, North-West University (Potchefstroom Campus); 2 Department of Social Peadiatrics, Hacettepe University, Ankara, Turkey; 3 Institute of Public Health, Hacettepe University, Ankara, Turkey

**Keywords:** Pre-lacteal feeds, mothers, infants, urban, rural, Nigeria

## Abstract

**Background:**

Prelacteal feeding (PLF) is a barrier to exclusive breast feeding.

**Objective:**

To determine factors associated with PLF in rural and urban Nigeria.

**Methods:**

We utilized data from the 2013 Nigerian Demographic and Health Survey. Bivariate and multivariate analyses were used to test for association between PLF and related factors.

**Results:**

Prevalence of PLF in urban Nigeria was 49.8%, while in rural Nigeria it was 66.4%. Sugar or glucose water was given more in urban Nigeria (9.7% vs 2.9%), plain water was given more in rural Nigeria (59.9% vs 40.8%). The multivariate analysis revealed that urban and rural Nigeria shared similarities with respect to factors like mother's education, place of delivery, and size of child at birth being significant predictors of PLF. Mode of delivery and type of birth were significant predictors of PLF only in urban Nigeria, whereas, mother's age at birth was a significant predictor of PLF only in rural Nigeria. Zones also showed variations in the odds of PLF according to place of residence.

**Conclusion:**

Interventions aimed at decreasing PLF rate should be through a tailored approach, and should target at risk sub-groups based on place of residence.

## Background

Exclusive breast feeding (EBF) from birth through six months of age has long-term health and emotional benefits for both mother and child and is associated with lower infant morbidity and mortality as well as better growth^1^. Also, provision of mother's breast milk to infants within one hour of birth ensures that the infant receives colostrum which is rich in immunoglobulin (Ig) and other bioactive molecules important for nutrition, growth and for passive immunity^2^.

The World Health Organisation (WHO) and the United Nations Children's Fund (UNICEF) during the Innocenti Declaration in 1990 called for policies that would cultivate breast feeding culture and encourage women to breastfeed their infants exclusively for the first six months of life^3^.

Among the 10 steps to successful breast feeding is giving infants no food or drink other than breast milk, unless medically indicated^3^. Pre-lacteal feeds are foods given to newborns before breast feeding is established or before breast milk comes in^4^. Studies have shown that introducing these pre-lacteal feeds has the following negative effects; delaying breast feeding initiation, interfering with EBF, disrupting the mother-baby dyad, interfering with suckling, and exposing the baby to risk of infection^5^–^8^. In addition, pre-lacteal feeds have fewer nutrients and immunological components as compared to breast milk^9^.

Nigeria became a fully independent country in October 1960^10^. The population of Nigeria is estimated to be 182 million as of 2015 and the total health expenditure in 2014 was 3.7% of its Gross Domestic Product (GDP)^11^. In Nigeria, neonatal, infant and child mortality as well as malnutrition continue to be major health issues affecting the country^10^. Nigeria's neonatal mortality rate stands at 37 deaths per 1,000 live births while the infant mortality rate stands at 69 deaths per 1,000 live births^10^. However, despite these rates, studies have observed that the core indicators of optimal breast feeding in Nigeria are still low with only about 34.7 % of children initiating breast-feeding early and 17.4 % of infants under-five months of age being exclusively breast fed^1^,^12^.

Previous literature has shown that the determinant of PLF are multi-factorial in nature and includes factors such as mode of delivery, type of birth, occupation, education, place of delivery, size at birth, and regions^5^–^7^,^9^,^13^. Studies done in India and Malawi observed rural-urban differences in PLF prevalence with the prevalence of PLF reportedly being higher in rural areas as compared to urban areas^14^,^15^.

Breast feeding practices such as Early Initiation of Breast Feeding (EIBF) and EBF are the key and easiest intervention to reducing child death and morbidity^1^–^2^. An understanding of factors associated with PLF is important in the promotion of EBF and EIBF. In Nigeria, most previous research with regards to PLF has been based on nationally non-representative samples and these studies have been limited in their ability to compare urban and rural differences in PLF practice. This research fills this gap by examining a nationally representative sample to determine factors associated with PLF in rural and urban residence. This study aims to examine prevalence of PLF, types of pre-lacteal feeds and the determinants of PLF in urban and rural Nigeria. We hypothesized that the factors influencing PLF differ between urban and rural areas in Nigeria.

## Methods

### Study setting and ethics

This was a cross-sectional study using nationally representative data from the 2013 Nigeria Demographic and Health Survey (NDHS) and authorization to use the data was given by Measure DHS. The 2013 NDHS was implemented by the National Population Commission and it is the fifth in the series of Demographic and Health Surveys conducted so far in Nigeria. NDHS have the approval of the National Health Research Ethics Committee.

Administratively, Nigeria is divided into 36 states, and the Federal Capital Territory, Abuja. Each state is sub-divided into local government areas (LGAs), and each LGA is divided into localities.

### Sample

The sample for the 2013 NDHS was a stratified sample, selected independently in three stages. Stratification was achieved by separating each state into urban and rural areas. In the first stage, 893 localities were selected with probability proportional to size. In the second stage, one cluster was selected by simple random sampling. In a few larger localities, more than one cluster was selected. In total, 904 clusters (372 in urban areas and 532 in rural areas.) were selected. In the third stage of selection, a fixed number of 45 households were selected in every urban and rural cluster through equal probability systematic sampling.

All women aged 15–49 years who were either permanent residents of the households in the 2013 NDHS sample or visitors present in the households on the night before the survey were eligible to be interviewed. Three sets of validated questionnaires were utilized to collect data and included; a household questionnaire, a woman's questionnaire and a man's questionnaire.

A four-week-long training course in January and February 2013 was conducted for the field staff and the fieldwork was conducted from February 15, 2013, to the end of May (with the exception of the two teams in Kano and Lagos, who completed fieldwork in June).

In the interviewed households, a total of 39,902 women aged 15–49 years (Urban= 15,972 and rural = 23,930 women) were identified as eligible for individual interviews, and 98 percent of them were successfully interviewed^10^.

Analysis for this study was restricted to last-born ever breastfed children born in the past two years preceeding the survey and the total sample size was 3879 for urban and 7888 for rural residence. After accounting for sample weights, this corresponded to a sample size of 4172 for urban and 7637 for rural areas.

### Outcome variable

In the NDHS woman's questionnaire, mothers were asked “In the first three days after delivery, was (NAME) given anything to drink other than breast milk? What was (NAME) given to drink? (Options were: milk (other than breast milk); plain water; sugar or glucose water, gripe water, sugar salt water solution; fruit juice; infant formula; tea infusion; coffee, honey; and/others)^10^. Our outcome variable pre-lacteal feeding was defined as having given anything to drink other than breast milk in the first three days after delivery. The types of pre-lacteal feeds were reported as frequencies and percentages.

### Independent variables

The explanatory factors were chosen based on previous studies^5^–^7^,^9^,^13^,^14^ and grouped into two categories namely; maternal socio-demographic factors and antenatal and postnatal factors.

### Explanatory variables included the following;

(i) *Maternal socio-demographic factors*; Ungrouped mothers age at birth was recoded into <=19, 20–24, 25–29, 3034 and >=35 years. Mother's education was categorized as no education, primary, secondary and above. Mother's occupation was re-grouped into not working and working. DHS wealth index was categorized into lowest (poorest), second (poorer), middle, fourth (richer) and highest (richest) wealth quintile, the index was constructed using household asset data via a principal components analysis. All the six geopolitical zones were included in the study.

(ii) *Antenatal and postnatal factors;* We created a new variable combined birth interval and birth rank to compare the effect of birth order and subsequent birth interval with PLF, this variable was categorized into 5 categories namely; 1^st^ birth rank, 2^nd^–3^rd^ birth rank and preceding birth interval < =23 month, 2^nd^–3^rd^ birth rank and preceding birth interval 24 month and above, 4^th^ and above birth rank and preceding birth interval < =23 months, 4^th^ and above and preceding birth interval 24 month and above. Number of antenatal care (ANC) visits was recoded into 0, 1–3, 4 and above visit. Place of delivery was categorized as home and health facility. Also considered was mode of delivery (spontaneous vaginal or caesarean-section). Birth type was recoded into singleton or twin/multiple, sex of child was as reported in the 2013 NDHS (male-female), size of child at birth based on mothers perception (subjective birth weight) and was categorized into three groups namely; large, average and small.

### Statistical analysis

Chi square tests were performed to evaluate the association of the independent variables with PLF. Rate of PLF and distribution by different independent variables were reported as weighted percentages and 95 % CI using Stata version 13.0 (Stata Corp, College Station, TX, USA). Before running the multivariate analysis, we examined the correlation between explanatory variables that had high potential for collinearity. Binary logistic regression was used to examine the likely predictors of PLF in Nigeria. Factors considered for the multivariable model were based from previous literature. The logistic regression analysis consisted of 2 models. Model 1 was the maternal socio-demographic model while model 2 included model 1+ antenatal and postnatal factors. Adjusted odds ratios (AOR) with their 95% confidence interval (CI) were reported. The multivariate analysis accounted for the sample design and sample weight using Statistical Package for Social Sciences (SPSS) complex sample analysis method (SPSS version 21).

## Results

### Characteristics of the sample disaggregated by urban-rural residency

A higher proportion of urban and rural mothers at the time of birth were within the ages of 25–29 years (30.4% and 25.8%, respectively). 60.0% of urban mothers had secondary and above education, on the other hand, 57.8% of rural mothers had no education. The percentages of male and female children were more or less equal in both settings (Table 1).

**Table 1:** Characteristics of mother-baby pair sample (Nigeria, DHS 2013)

	Urban						Rural					
		
Characteristics	Total		Children who received PLF				Total		Children who received PLF			
	N †	% ¶	n §	% ‡	95%CI	P value	N †	% ¶	n§	% ‡	95%CI	P value
***Maternal socio demographic,*** ***characteristics***												
Mother's age at birth												
<=19	317	8.2	217	63.5	(58.4–68.6)	<0.001	1336	16.9	1019	73.7	(71.4–76.0)	<0.001
20–24	931	24.0	507	51.6	(48.5–54.7)		1998	25.3	1248	64.7	(62.5–66.8)	
25–29	1181	30.4	630	48.4	(45.7–51.1)		2035	25.8	1256	64.2	(62.1–66.4)	
30–34	820	21.1	387	44.2	(41.0–47.5)		1282	16.3	761	63.0	(60.3–65.7)	
>=35	630	16.2	334	49.9	(46.1–53.7)		1237	15.7	788	67.8	(65.1–70.5)	
Mother's education												
No education	774	20.0	553	64.3	(61.1–67.5)	<0.001	4557	57.8	3596	75.7	(74.5–76.9)	<0.001
Primary	774	20.0	427	52.6	(49.2–56.0)		1502	19.0	731	55.8	(53.1–58.4)	
Secondary and above	2331	60.0	1096	43.8	(41.9–45.8)		1829	23.2	745	47.3	(44.8–49.8)	
Mother's occupation												
Non-working	1042	27.0	638	56.3	(53.4–59.1)	<0.001	2645	33.7	1780	68.9	(67.1–70.7)	0.001
Working	2820	73.0	1427	47.3	(45.5–49.0)		5200	66.3	3272	65.1	(63.8–66.4)	
Wealth index												
Lowest	144	3.7	93	64.6	(56.9–72.6)	<0.001	2455	31.1	1995	77.0	(75.3–78.6)	<0.001
Second	269	6.9	167	56.2	(50.6–61.9)		2463	31.2	1583	66.2	(64.2–68.0)	
Middle	677	17.5	389	58.3	(54.5–62.0)		1675	21.2	911	58.3	(55.9–60.8)	
Fourth	1216	31.3	686	52.3	(49.6–55.0)		975	12.4	467	57.7	(54.3–61.1)	
Highest	1573	40.6	741	42.3	(40.0–44.6)		320	4.1	116	41.3	(35.6–47.1)	
Zones												
North Central	530	13.7	146	37.6	(32.8–42.4)	<0.001	1204	15.3	673	54.3	(51.5–57.1)	<0.001
North East	505	13.0	321	63.6	(59.4–67.8)		1905	24.2	1217	79.6	(77.6–81.6)	
North West	684	17.6	629	67.6	(64.6–70.6)		2963	37.6	2428	72.3	(70.8–73.8)	
South East	688	17.7	405	55.0	(51.4–58.6)		382	4.8	193	56.8	(51.5–62.0)	
South South	437	11.3	161	41.4	(36.5–46.3)		989	12.5	375	51.6	(48.0–55.2)	
South West	1035	26.7	414	33.9	(31.2–36.5)		445	5.6	185	41.8	(37.2–46.4)	
***Antenatal and postnatal factors***												
Combined birth interval and rank												
1st birth rank	869	22.4	485	51.7	(48.4–54.9)	<0.001	1447	18.4	947	66.5	(64.1–67.0)	0.052
2nd–3rd birth rank,<=23 months interval	324	8.4	177	50.3	(45.1–55.5)		445	5.8	263	63.2	(58.6–67.9)	
2nd–3rd birth rank, 24 months and above interval	1079	27.9	498	43.8	(40.9–46.7)		1904	24.2	1222	64.7	(62.5–66.8)	
4^th^ and above birth rank,<=23 months interval	253	6.5	149	56.9	(50.7–62.7)		618	7.9	380	64.5	(60.7–68.4)	
4th and above birth rank, 24 months and above interval	1346	34.8	757	51.4	(48.9–60.0)		3445	43.8	2246	68.0	(66.4–69.6)	
Antenatal care visit												
0	384	10.3	268	63.1	(58.4–67.6)	<0.001	3421	44.2	2539	73.2	(71.8–74.7)	<0.001
1–3	432	11.6	276	57.4	(52.9–61.7)		1130	14.6	727	67.4	(64.6–70.2)	
4 and above	2917	78.1	1477	47.3	(45.6–49.1)		3187	41.2	1737	59.0	(57.2–60.7)	
Place of delivery												
Home	1337	34.5	930	61.6	(59.1–64.0)	<0.001	5937	75.4	4245	72.5	(71.3–73.6)	<0.001
Health facility	2535	65.5	1145	43.1	(41.2–45.0)		1934	24.6	821	46.5	(44.1–48.8)	
Mode of delivery												
Spontaneous vaginal delivery	3637	95.4	1964	50.0	(48.5–51.6)	0.238	7789	98.9	5025	66.6	(65.5–67.7)	0.009
Caesarean section	175	4.6	92	54.8	(47.2–62.3)		87	1.1	43	52.4	(41.4–63.2)	
Type of birth												
Single	3814	98.3	2027	49.4	(47.9–51.0)	<0.001	7759	98.4	4981	66.4	(65.3–67.4)	0.782
Multiple	65	1.7	49	73.1	(61.8–83.4)		129	1.6	91	67.9	(59.8–75.7)	
Sex of child												
Male	1963	50.6	1027	49.4	(47.2–51.5)	0.642	4010	50.8	2566	66.9	(65.4–68.4)	0.370
Female	1916	49.4	1048	50.1	(48.0–52.3)		3878	49.2	2506	65.9	(64.4–67.4)	
Size of child at birth												
Small	478	12.4	303	58.3	(53.9–62.4)	<0.001	1321	16.8	964	75.1	(72.7–77.4)	<0.001
Average	1604	41.5	939	53.3	(51.0–55.7)		3141	40.1	2133	70.7	(69.0–72.3)	
Large	1784	46.1	828	44.1	(41.8–46.3)		3379	43.1	1952	59.2	(57.5–60.9)	

† Unweighted case numbers,

¶ Column %,

§ Weighted case numbers,

‡ Row %.

### Prevalence of pre-lacteal feeds and types disaggregated by urban-rural residency

**FIG. 1:**
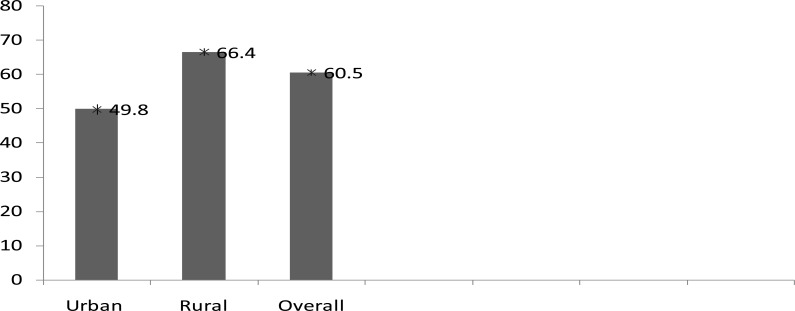
Prevalence of pre-lacteal feeding in Nigeria (Nigeria, DHS 2013).

The overall prevalence of PLF in Nigeria was 60.5% (95 % CI: 59.6%–61.4%). The prevalence of PLF observed in urban area was 49.8%, (95 % CI: 48.2%–51.3%). while in rural areas it was 66.4% (95 % CI: 65.3%–67.5%) (Fig. 1).

**FIG. 2:**
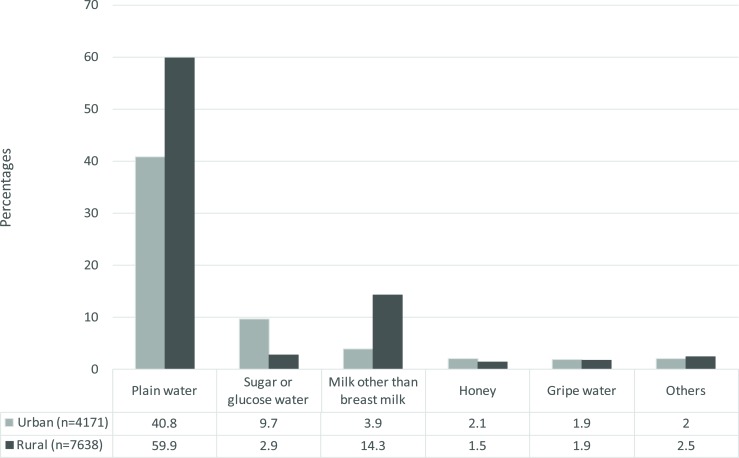
Types of prelacteal feed given among last-born children under two years of age (Nigeria, DHS 2013) Note: Others category include any of the following prelacteal feeds; sugar/salt solution, fruit juice, infant formula, tea/infusions, coffee, or other.

Sugar or glucose water (9.7 vs 2.9%) and honey (2.1 vs 1.5%) were predominatly given in urban Nigeria, whereas plain water (59.9 vs 40.8%), milk other than breast milk (14.3% vs 3.9%) and other pre-lacteal feeds (2.5 vs 2%) were commonly given in rural Nigeria. Gripe water was evenly given in both urban and rural Nigeria (Fig. 2).

### Bivariate results

**Urban Nigeria:** In urban Nigeria, the explanatory variables that were significantly associated with higher PLF rates included: Mothers age at birth being <=19 years, no education, non-working mothers, belonging to the lowest wealth quartile, all geopolitical zones as compared to the South Western zone, 4^th^ birth rank and above with preceding birth interval of less than or equal to 23 months, no ANC visits, home delivery, multiple births, and small size of baby at birth (Table 1).

**Rural Nigeria:** In rural Nigeria, the significant covariates associated with higher PLF rates included; Mother's age at birth being <=19 years, no education, non-working mothers, belonging to the lowest wealth quintile, all geopolitical zones as compared to the South West zone, no ANC visits, home delivery, spontaneous vaginal delivery and small size of child at birth. (Table 2).

**Table 2:** Determinants of PLF in urban-rural Nigeria (Nigeria, DHS 2013)

	Urban						Rural					
		Model 1 †			Model 2 ¶			Model 1 †			Model 2 ¶	
	
Characteristics	AOR	(95%CI)	p	AOR	95%CI	p	AOR	(95%CI)	p	AOR	95%CI	p
***Maternal sociodemographic*** ***characteristics***												
Mother's age at birth												
<=19	**1.61**	**(1.13–2.28)**	**0.008**	1.42	(0.95–2.12)	0.092	**1.42**	**(1.16–1.73)**	**0.001**	**1.35**	**(1.02–1.79)**	**0.037**
20–24	1.08	(0.83–1.42)	0.569	1.09	(0.80–1.48)	0.592	1.03	(0.88–1.22)	0.686	1.04	(0.85–1.28)	0.682
25–29	1.07	(0.82–1.39)	0.638	1.03	(0.77–1.37)	0.857	0.95	(0.81–1.12)	0.553	0.98	(0.82–1.17)	0.800
30–34	0.90	(0.70–1.16)	0.413	0.93	(0.72–1.21)	0.598	0.91	(0.75–1.10)	0.317	0.91	(0.75–1.12)	0.367
>=35	1.00			1.00			1.00			1.00		
Mother's education												
No education	**1.53**	**(1.13–2.07)**	**0.006**	**1.48**	**(1.07–2.04)**	**0.017**	**2.95**	**(2.30–3.78)**	**<0.001**	**2.71**	**(2.11–3.48)**	**<0.001**
Primary	**1.33**	**(1.04–1.69)**	**0.022**	**1.31**	**(1.02–1.69)**	**0.037**	**1.40**	**(1.15–1.71)**	**0.001**	**1.34**	**(1.08–1.66)**	**0.008**
Secondary and above	1.00			1.00			1.00			1.00		
Mother's occupation												
Non-working	1.00			1.00			1.00			1.00		
Working	0.87	(0.71–1.06)	0.162	0.88	(0.72–1.09)	0.244	1.14	(0.97–1.33)	0.107	1.13	(0.97–1.32)	0.128
Wealth index												
Lowest	1.00			1.00			1.00			1.00		
Second	0.79	(0.47–1.32)	0.364	0.78	(0.45–1.35)	0.369	0.80	(0.66–0.98)	0.028	0.84	(0.69–1.03)	0.099
Middle	0.99	(0.68–1.44)	0.944	1.05	(0.70–1.58)	0.821	0.88	(0.67–1.14)	0.335	0.98	(0.74–1.31)	0.906
Fourth	1.05	(0.74–1.51)	0.772	1.16	(0.77–1.73)	0.478	1.18	(0.86–1.60)	0.304	1.38	(0.99–1.91)	0.053
Highest	1.03	(0.70–1.53)	0.869	1.11	(0.72–1.72)	0.646	0.73	(0.50–1.07)	0.103	0.93	(0.62–1.38)	0.714
Zones												
North Central	1.06	(0.73–1.54)	0.778	1.04	(0.70–1.54)	0.845	1.49	(0.96–2.33)	0.076	1.45	(0.95–2.21)	0.089
North East	**2.69**	**(1.88–3.86)**	**<0.001**	**2.63**	**(1.78–3.89)**	**<0.001**	**3.76**	**(2.33–6.05)**	**<0.001**	**3.25**	**(2.05–5.16)**	**<0.001**
North West	**3.37**	**(2.31–4.90)**	**<0.001**	**3.20**	**(2.13–4.81)**	**<0.001**	**2.22**	**(1.42–3.47)**	**0.001**	**1.83**	**(1.19–2.82)**	**0.006**
South East	**2.41**	**(1.74–3.32)**	**<0.001**	**2.19**	**(1.56–3.08)**	**<0.001**	**2.67**	**(1.53–4.65)**	**0.001**	**2.72**	**(1.55–4.75)**	**<0.001**
South South	1.38	(0.99–1.91)	0.056	1.31	(0.89–1.93)	0.177	**1.88**	**(1.21–2.91)**	**0.005**	**1.66**	**(1.08–2.55)**	**0.021**
South West	1.00			1.00			1.00			1.00		
***Antenatal and postnatal*** ***factors***												
Combined birth interval and rank												
1st birth rank				1.25	(0.94–1.67)	0.129				1.11	(0.86–1.45)	0.426
2nd–3rd birth rank <=23 months interval				1.03	(0.76–1.39)	0.871				1.00	(0.74–1.34)	0.983
2nd–3rd birth rank, 24 months and above interval				0.94	(0.73–1.21)	0.627				0.99	(0.83–1.19)	0.948
4th birth rank <=23 months interval				1.20	(0.87–1.67)	0.264				0.82	(0.65–1.04)	0.095
4th birth rank, 24 months and above interval				1.00						1.00		
Antenatal care visit												
None				0.84	(0.61–1.16)	0.290				0.91	(0.76–1.08)	0.280
1–3				0.84	(0.62–1.15)	0.285				0.96	(0.78–1.19)	0.722
4 and above				1.00						1.00		
Place of delivery												
Home				**1.53**	**(1.24–1.89)**	**<0.001**				**2.05**	**(1.72–2.43)**	**<0.001**
Health facility				1.00						1.00		
Mode of delivery												
Spontaneous vaginal delivery				1.00						1.00		
Caesarean section				**1.87**	**(1.25–2.80)**	**0.003**				1.21	(0.68–2.15)	0.511
Type of birth												
Single				1.00						1.00		
Multiple				**2.37**	**(1.14–4.95)**	**0.022**				1.20	(0.75–1.94)	0.449
Size of child at birth												
Small				**1.46**	**(1.10–1.94)**	**0.009**				**1.77**	**(1.44–2.17)**	**<0.001**
Average				**1.57**	**(1.27–1.93)**	**<0.001**				**1.66**	**(1.45–1.91)**	**<0.001**
Large				1.00						1.00		
Sex of child												
Male				1.02	(0.85–1.21)	0.863				1.07	(0.96–1.21)	0.227
Female				1.00						1.00		

*Nagelkarke Pseudo R* *Square*	0.114			0.141			0.127			0.160		

† Model 1 = Maternal sociodemographic characteristics,

¶ Model 2= Model1 + Antenatal and postnatal factors.

### Multivariate results

**Urban Nigeria:** When maternal socio-demographic, antenatal and postnatal factors were controlled for (Model 2), urban mothers with no education and primary educational status had significantly 48 and 31% higher odds of PLF as compared to mothers with secondary and above educational status (Adjusted Odd Ratio (AOR)=1.48, 95% CI=1.07–2.04 and AOR=1.31, 95% CI=1.02–1.69, respectively). Also, compared to the South Western geopolitical zone, urban mothers, who lived in the following geopolitical zones of Nigeria, were significantly more likely to give prelacteal feeds: North East (AOR=2.65, 95% CI=1.78–3.89); North West (AOR=3.20, 95% CI=2.13–4.81); and South East (AOR=2.19, 95% CI=1.56–3.08). Urban mothers who delivered at home had significantly higher odds of PLF as compared to urban mothers whose place of delivery were health facility (AOR=1.53, 95% CI=1.24–1.89). The odds of PLF was 1.87 times higher for urban mothers who had caesarean section as compared to urban mothers who had spontaneous vaginal delivery (AOR=1.87, 95% CI=1.25–2.80). In addition, urban mothers with multiple births had a 2.37 times the odds of PLF as compared to mothers with singleton birth (AOR= 2.37, 95% CI=1.14–4.95). We further observed that urban mothers who perceived the size of their child at birth to be small or average had significantly higher odds for PLF as compared to urban mothers who perceived the size of their child at birth to be large (AOR=1.46, 95% CI=1.10–1.94 and AOR=1.57, 95% CI=1.27–1.93, respectively).

**Rural Nigeria:** In rural Nigeria, Model 2 showed that mothers who were aged <=19 years at birth were significantly more likely to give pre-lacteal feeds as compared to those aged 35 years and above at birth (AOR=1.35, 95% CI=1.02–1.79). Also, rural mothers with no education (AOR=2.71, 95% CI=2.11–3.48) and primary educational status (AOR=1.34, 95 % CI=1.08–1.66) were significantly more likely to give pre-lacteal feeds as compared to mothers with secondary and above educational status. Compared to the South Western geopolitical zone, rural mothers, who lived in the following geopolitical zones of Nigeria, were significantly more likely to give prelacteal feeds: North East (AOR=3.25, 95% CI=2.05–5.16); North West (AOR=1.83, 95% CI= 1.19–2.82); South East (AOR=2.72, 95% CI=1.55–4.75) and South South (AOR=1.66, 95% CI=1.08–2.54). The odds of PLF was 2.05 times higher for rural mothers who delivered at home as compared to mothers who delivered in a health facility (AOR=2.05, 95% CI= 1.72–2.43). Rural mothers who perceived their babies as small (AOR=1.77, 95 CI=1.44–2.17) or average sized (AOR 1.66, 95% CI=1.45–1.91) at birth were more likely to give pre-lacteal feeds as compared to rural mothers who perceived their child to be large at birth.

## Discussion

The main finding of this study was that PLF practice was more common in rural Nigeria (66.4%) as compared to urban Nigeria (49.8%). Prevalence of PLF was also higher in rural as compared to urban areas in India and Malawi^14^–^15^. The observed difference in PLF prevalence between urban and rural areas may be explained by the fact that urban areas differ socio-culturally from rural areas in many ways and such differences at both individual, household and community levels may play a role^16^.

The commonest prelacteal feeds in Nigeria were plain water, sugar or glucose water and milk other than breast milk. This agrees with previous studies done in countries like Kenya^17^, Philippines^18^, and Nepal^7^. We also observed that sugar or glucose-water and honey were given more in urban Nigeria, whereas, plain water, milk other than breast milk and other pre-lacteal feeds were given commonly more in rural Nigeria. We postulated that the variation between urban and rural areas in the types of pre-lacteal feeds could be attributed to the availability of different feeds and/or cultural differences in both settings.

In the full model, urban and rural Nigeria shared similarities with respect to factors like mothers education, place of delivery, and size of child at birth being significant predictors of PLF, however some factors such as mode of delivery, type of birth, and mother's age at birth, showed variation in terms of significance according to place of residence. Mode of delivery, and type of birth were significant predictors of PLF only in urban Nigeria, whereas, mother's age at birth was a significant predictors of PLF only in rural Nigeria. Zones also showed variations in the odds of PLF according to place of residence. In urban Nigeria, caesarean section contributed significantly to a higher likelihood of PLF. The high rates of PLF among women who had caesarean section as compared to spontaneous vaginal deliveries could be linked to the fact that caesarean section (CS) is associated with prolonged maternal-infant separation, antibiotics safety concern on the child, pain and discomfort, and longer stay in the hospital^19^. However, we observed a lack of significance between caesarean section and PLF in rural Nigeria, though, rural mothers who had CS were more likely to give pre-lacteal feeds as compared to rural mothers who had spontaneous vaginal delivery. The 2013 NDHS reported the prevalence of caesarean section to be 1.0 % in rural areas as compared to 3.9 % in urban areas^10^.

The result of this study showed that urban mothers who had multiple births were more likely to give PLF as compared to urban mothers who had singleton births. This result is in consonance with findings of a previous study that report that establishment of breastfeeding after multiple births is extremely difficult^20^. Another study reported the following reasons for breast feeding among mothers with multiple births; mother simply did not want to breast feed, maternal or infant illness, physician advice against insufficient milk supply, and not enough time^21^. In the case of rural Nigeria, rural mothers with multiple where also more likely to give pre-lacteal feed as compared to rural mothers with singleton birth, however, this finding was not a significant finding for rural Nigeria. We postulated that the major reason for the lack of significance among rural mothers was that rural mothers who had multiple births have lower access to expensive infant feeding alternatives as compared to urban mothers. On the other hand, this argument alone cannot explain the lack of significance observed in rural Nigeria and raises the need for further investigation.

In rural Nigeria, mothers aged less than or equal to 19 years were significantly more likely to offer pre-lacteal feed as compared to older mothers aged 35 years and above. The reason could be that younger mothers may lack knowledge or experience about appropriate breastfeeding practices^22^. In urban Nigeria, this finding was true controlling only for other maternal socio-demographic characteristics. However, this significance was lost when antenatal and postnatal variables were controlled for.

Maternal education was an important determinant of PLF, although not strongly so, in both settings. The odds of PLF were higher for mothers with no education or primary education as compared to mothers with secondary and above educational status. A probable reason could be that the longer time spent in formal education put mothers in a better position to self-educate themselves on infant nutrition^23^.

Our study findings showed that place of delivery was significantly associated with PLF practice in both urban and rural Nigeria. Mothers who delivered at home were more likely to give pre-lacteal feeds as compared to mothers who delivered in a health facility. This is in consonance with a study done in Ethopia^24^. These findings could be as a result of the fact that mothers who deliver in the hands of health personnel's were more likely to be encouraged and counseled for healthy infant feeding practices. In the Nigerian context, our result was not a surprising finding as many of the primary health care centers and hospitals in Nigeria have adopted the Baby Friendly Hospital Initiative (BFHI) and the policy in these health care facilities is for the midwife or any other available skilled provider to give newborn infants no food or drink other than breast milk, unless medically indicated^25^.

Mothers in both urban and rural Nigeria, mothers who perceived their infant to be small or average sized at birth were more likely to introduce pre-lacteal feeds as compared to mothers who perceived their infants to be large sized. In consonance, Flaherman and colleagues found that higher birth weight was strongly associated with exclusive breastfeeding^26^ while Berde and Yalcin^12^ reported that larged sized infant had higher likelihood of EIBF. Flaherman and collegues suggested that mothers of smaller sized infants might worry more about infant weight and about milk supply, possibly leading to unnecessary formula supplementation^26^.

The current study observed significant zonal variations in PLF odds in both urban and rural Nigeria. Regional differences in PLF in both urban and rural Nigeria could be in part a function of access to health service, inequitable distribution of health services, health information, resources and other geographic differences, as shown in another study^7^. In addition, cultural practices may play a role and this role has been observed to vary across different settings^7^,^27^–^31^.

This study is not without some limitations, the study limitation relates to the fact that the data was based on a cross-sectional study and is subject to recall bias. In addition, due to the cross-sectional nature of the data, caution must be exercised in making causal influence of the identified determinants of PLF. On the other hand, the study has strength of being a nationally representive study with a high response rate, in addition, complex sample analysis was performed to account for the sampling strategy and sample weight, thus, the findings are generalizable to the entire country. Future studies using qualitative approaches such as in-depth interview of some key informants will help in enriching the knowledge on PLF in Nigeria.

## Conclusion

We observed differences in PLF between urban and rural areas, with factors affecting PLF showing variation in terms of significance according to place of residence. Interventions aimed at decreasing PLF rate should be through a tailored approach, targeting at risk sub groups discovered in our study.
